# The Acute Effect of Dynamic vs. Proprioceptive Neuromuscular Facilitation Stretching on Sprint and Jump Performance

**DOI:** 10.3390/jfmk9010042

**Published:** 2024-02-28

**Authors:** Nor Fazila Abd Malek, Ali Md Nadzalan, Kevin Tan, Abdul Muiz Nor Azmi, Rajkumar Krishnan Vasanthi, Ratko Pavlović, Dana Badau, Adela Badau

**Affiliations:** 1 Faculty of Sport Science and Coaching, Sultan Idris Education University, Tanjong Malim 35900, Perak, Malaysia; fazila.malek@fsskj.upsi.edu.my (N.F.A.M.); ali.nadzalan@fsskj.upsi.edu.my (A.M.N.); 2 Faculty of Psychology and Education, University Malaysia Sabah, Jalan UMS, Kota Kinabalu 88400, Sabah, Malaysia; k.tan@lboro.ac.uk (K.T.); abdulmuiz@ums.edu.my (A.M.N.A.); 3 School of Sport, Exercise and Health Sciences, Loughborough University, Epinal Way, Loughborough LE11 3TU, UK; 4 Faculty of Health and Life Science, INTI International University, Nilai 71800, Negeri Sembilan, Malaysia; rajkumar.krishnan@newinti.edu.my; 5 Faculty of Physical Education and Sport, University of East Sarajevo, Vuka Karadzica 30, 71126 East Sarajevo, Bosnia and Herzegovina; pavlovicratko@yahoo.com; 6 Faculty of Physical Education and Mountain Sports, Transilvania University, 500068 Brasov, Romania; adela.badau@unitbv.ro

**Keywords:** stretching, warm-up, strength and conditioning, training protocol

## Abstract

Participating in sports has been shown to promote overall wellness and, at the same time, reduce health risks. As more people are participating in sports, competitions have increased, and every aspect of the game has been focused by coaches and athletes in order to improve performance. One of these aspects is the warm-up session. The purpose of this study was to investigate the acute effect of a dynamic warm-up versus a proprioceptive neuromuscular facilitation (PNF) warm-up on the sprint and jump performance of recreationally active men. Thirty (n = 30) males were randomly assigned to undergo three sessions of different warm-up types, 72 h apart, involving either proprioceptive neuromuscular facilitation (PNF), dynamic stretching (DS), or no stretching session (control). The PNF and dynamic modes of stretching improved vertical jump performance, F (2.58) = 5.49, *p* = 0.046, to a certain extent (mean + 3.32% vs. control, *p* = 0.002 for dynamic and mean + 1.53% vs. control, *p* = 0.048 for PNF stretching). Dynamic stretching is best used to get a better vertical jump height. Sprint performance was also increased to a greater extent following the stretching session, F (2.58) = 5.60, *p* = 0.01. Sprint time was +1.05% faster vs. the control, with a value of *p* = 0.002 after dynamic stretching, while PNF stretching demonstrated a sprint time of +0.35% vs. the control, with a value of *p* = 0.049. Dynamic stretching showed a better sprint performance and also vertical jump height performance in this study. PNF and dynamic stretching prove to be equally efficacious in flexibility conditioning depending on the type of movement involved. This type of stretching should be utilized to help preserve or improve the performance output of physical activity, especially in sprinting and jumping events.

## 1. Introduction

Stretching prior to exercise is a norm among all athletes and the recreational population. It is a way for us to prepare our body for physical activity, training, or any sporting event by improving joint range of motion and muscle elasticity. Doing so can improve physical activity in terms of performance output and also reduce the chances of injury [1]. Consequently, it may also result in increased body core temperature in preparation for activities [1,2]. Coaches and athletes often include stretching exercises as a part of their training program or as a pre-event warm-up activity [3]. Stretching falls under the physical health components of flexibility. Flexibility, on the other hand, affects muscular performance [4]. It is recommended to do a stretching exercise for a healthy recreational population or athletes to prevent injury, for rehabilitation, and to increase athletic performance [5]. It was also suggested that the development of performance in the long term is related to an increase in stretching ability during activity and the tendency of a muscle to move in a flexible and less stiff way, which is an ideal condition for a resistance exercise to take place [6].

However, despite all the benefits, certain articles have speculated about the other proposed benefits stretching has to offer concerning the widespread acceptance and use of stretching [7,8,9]. Stretching, especially static or passive stretching, has been found in several studies to cause a considerable acute decline in various maximal muscle performances, such as force or power output [2,10,11,12,13], vertical jump performance [14,15], and sprinting performance [16]. These effects have ramifications for athletes who participate in power-based sports activities that demand strength and power generation, such as gymnastics, football, and sprinting, prompting some studies to advise avoiding static stretching before such events.

To overcome this issue, fitness enthusiasts, strength and conditioning coaches, and sports scientists have turned their attention to forming a combination form of stretching that could be utilized without a significant decrease in performance output. Various forms of active or dynamic warm-ups that incorporate movement while stretching have been designed and applied to prepare athletes during warm-up sessions [17]. Previous studies have shown that engaging in dynamic stretching exercises can lead to enhancements in physical activity performance, specifically in areas such as vertical jump performance and leg extension power [18,19]. Numerous studies also demonstrated an acute increase in power, sprint, or jump performance after dynamic stretches [20,21,22,23,24]. However, some studies contradict the idea that dynamic stretching is effective for performance development, such as by Nelson et al. [13], who found that dynamic stretching reduces the performance output of knee strength. This shows that a clear verdict on the effect of dynamic stretching has not been achieved.

Another type of stretching that is commonly used is proprioceptive neuromuscular facilitation (PNF) stretching. Static stimulation of the stretched muscle is used in PNF to produce optimal muscular relaxation [25]. Although studies on PNF are limited, there are interesting studies that have been obtained on the benefits of PNF stretching. PNF stretching is found to result in faster agility time because PNF has been shown to produce an increase in musculotendinous unit (MTU) stiffness [26]. An increase in muscular strength is also observed when PNF is done before sports practice with enough duration and consistency [27,28], and it also showed positive feedback on vertical jump performance [29]. Regardless, a study also indicated that PNF is shown to decrease performance before exercise with maximum effort [30,31,32] and it was also demonstrated to result in a negative effect when doing an isometric strength test [33].

Despite the acute effect of stretching on sports performance differing between types of stretching and movement, the PNF technique is considered to be one of the better methods to be utilized when compared with other stretching techniques, and studies not approving PNF are very few [34]. Furthermore, a significant portion of prior research has been focused on static stretching, with limited investigations conducted on the effects of two or more forms of stretching and their comparative outcomes.

This research endeavor is conducted with the aim of mitigating the persistent variability observed in various stretching protocols. This study also aims to add to the literature a comparison between techniques of stretching and their effects on physical activity performance. So, the purpose of this study is to see the immediate effect of PNF stretching and dynamic stretching on sprint and vertical jump performance among recreationally active individuals.

## 2. Materials and Methods

### 2.1. Participants

A total of thirty (n = 30) physically fit and active male individuals were recruited on a voluntary basis. The participants had an average age of 23.30 ± 3.33 years, a body height of 171.70 ± 2.84 cm, and a body mass of 76.43 ± 6.34 kg. Participants in this study involved male university students who had various sports backgrounds, whether recreational or competitive; they are also engaged in leisure-time physical activity at least three times per week. However, the requirement to be involved in this study does require participants to be experienced in jumping or sprinting [35,36]. In order to determine the required sample size for this study, a power analysis was conducted using G*Power software (version 3.1.9.4) for ANOVA repeated measures, within factors (small to medium effect size of 0.30, *p*-value of 0.05 and power of 0.80); the analysis revealed that a total sample size of n = 21 was adequate. Thus, n = 30 participants were recruited in this study, taking into account if any drop out occurred. They were randomly assigned to one of three experimental conditions, which included proprioceptive neuromuscular facilitation (PNF), dynamic stretching, and no stretching protocols. Prior to their involvement in this study, all participants were required to complete a Physical Activity Readiness Questionnaire (PAR-Q) and provide written informed permission. All individuals involved in the study were recreational sports players who engaged in physical activity three times per week. However, none of the participants had undergone any structured flexibility or strength training. The participants were devoid of any physical injuries during the assessment period and were given instructions to abstain from engaging in physically demanding activities for a duration of 24 h before the testing. The research study was carried out in adherence to the principles outlined in the Declaration of Helsinki, and the assessment methods were granted approval by the Ethics Committee for Human Testing at Sultan Idris Education University (Code: 2021-0442-01).

### 2.2. Study Design

**Figure 1:**
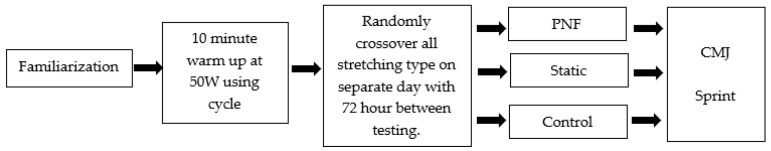
Flowchart of experiment protocol.

Indicate methods and the purpose of their use: Participants were ready to be tested on 3 separate days, with at least 72 h between testing days to allow for a full recovery. Based on previous studies [37,38], duration of 48 to 72 h was recommended recovery time between sessions that involve plyometric and strength training exercise. Since this study does not involve heavy exercise, 72 h between testing days should be adequate. In the first session, the participant was familiarized with all the procedures. During the familiarization, the participants performed 3 sprint trials and 3 vertical jump trials to reduce the likelihood of a learning effect during the study [29,39]. On each data collection day, all the participants are required to complete a 10 min aerobic warm-up at 50 W using a stationary cycle ergometer [39]. Two (2) minutes of rest were given after the aerobic warm-up session. Each subject was then randomly assigned to perform 1 of 2 stretching protocols (i.e., dynamic or PNF) or a no-stretch control condition. The orders of the stretch protocol conditions were systematically varied for the 30 subjects. This modification was implemented to mitigate the impact of order effects, enabling every participant to complete both stretching procedures (Protocol A and Protocol B) as well as the control condition during the designated testing sessions. After the stretching session, the participant is required to undergo the vertical jump test and sprint test to measure their performance. The time period between the vertical jump test and sprint test was 5 min. Figure 1 shows flowchart for experiment protocol.

### 2.3. Experimental Protocols

#### 2.3.1. PNF Stretching Protocols

The PNF stretching technique employed a “contract-relax-agonist-contraction” approach [40] necessitating the involvement of two individuals and requiring the subject to assume a supine position. The limb of choice was gradually and passively extended towards the maximum range of motion. The proprioceptive neuromuscular facilitation (PNF) stretch is commonly performed on the hamstring, gastrocnemius, gluteus, quadriceps, and hip flexor muscles. Subsequently, the participant endeavored to elicit maximal activation of the antagonist muscle groups associated with the favored limb, maintaining the leg in a fixed position for approximately 10 s. Following a brief 5 s period of rest, the participant is required to exert maximal activation of the agonist muscle groups. With the aid of the administrator, the limb is then maneuvered to achieve an enhanced range of motion (ROM) endpoint. The duration of this posture is 10 s [41,42]. This process is repeated another 2 times with 30 s of rest given between repetitions. PNF stretching involved in this study is shown in Table 1, consisting of (1) hamstring stretch, (2) quadricep stretch, (3) groin (butterfly) stretch, and (4) glute stretch. PNF implementation technique was based on studies from [43].

#### 2.3.2. Dynamic Stretching Protocol

The dynamic stretch encompasses the engagement and activation of muscles through the execution of rhythmic movements. The procedure involved the execution of a butt-kick exercise, wherein the participant repetitively and alternately brought the heel of each foot towards the buttocks while moving forward, with the objective of performing the exercise as swiftly as feasible. The procedure also involved the walking heel touch, where participants walk forward and touch their heel with both hands. The walking squat exercise involves the individual performing a squat at each stopping point while moving forward. The proposed exercise regimen includes walking lunges with a rotation, wherein the participant executes a substantial forward step while simultaneously performing a horizontal arm rotation. Additionally, the regimen incorporates a stretching exercise known as the hurdles leg raise, wherein participants ambulate with both hands extended anteriorly, palms facing downward, and proceed to elevate their extended leg towards the palm. The aforementioned technique was executed for a duration of 30 s on each occasion, with a total of 3 repetitions. Additionally, 20 s rest intervals between repetitions were given.

### 2.4. Measures

Vertical Jump Test. Participants stand with feet at shoulder width. Start in a standing position under the vertical jump equipment (Vertec, Sports Imports, Hilliard, OH, USA). Participants bend their knees and then jump vertically as high as possible, using both arms and legs to assist in projecting the body upwards. Participants try to reach the highest point possible on the Vertec equipment. The best of three attempts is recorded. The reliability of measurements obtained from the Vertec jumping equipment for vertical jump (VJ) height was assessed using intraclass correlation coefficients (ICCs). The ICC estimates and their corresponding 95% confidence intervals were calculated for three trials of VJ. The ICC estimates were as follows: ICC = 0.87, 95% CI = [0.43–0.87] for trial 1; ICC = 0.90, 95% CI = [0.85–0.95] for trial 2; and ICC = 0.92, 95% CI = [0.52–0.79] for trial 3. These results suggest good-to-excellent reliability [44] of the Vertec jumping equipment for measuring VJ height across multiple trials.

The 20 m Sprint Test. The experimental procedure entails the execution of a solitary maximal sprint covering a distance of 20 m, while employing a timing gate (Microgate, Bolzano, Italy) to accurately measure the duration of the sprint. The height of the timing gate was set at 1 m from ground due to the fact that the average height of adult male hip is at that height [45]. The participant assumes a standing split-stance start posture on the start line, with one foot positioned in front of the other. The position of the front foot is required to be situated posterior to the starting line. The initial position should be maintained for a duration of two seconds before commencing, and any swaying motions are prohibited. On the ‘Go’ signal, the participant must accelerate maximally to the finishing line. The reliability of sprint timing data obtained from timing gates was assessed using intraclass correlation coefficients (ICCs). Participants completed three sprint trials, and the timing data (in seconds) for each trial were recorded. The ICC estimates and standard errors of measurement (SEM) were calculated. Result of ICC = 0.92, SEM = 0.05 indicates excellent reliability [44] of the timing gates for measuring sprint times across the three trials.

### 2.5. Statistical Analysis

Data are reported as means and standard deviations. The assumption of normality was verified using the Shapiro–Wilk test. A repeated-measure analysis of variance (ANOVA) was used to compare the effects of different stretching types on sprint and vertical jump performance. A Bonferonni post-hoc test was applied to make a pairwise comparison between the data obtained when there was a significant effect detected. The statistical significance level for analyses was set at *p* < 0.05.

## 3. Results

Table 2 shows a test of the within-subject effect for vertical jumps. There is a significant effect in vertical jump performance among the three types of stretching protocol applied, F (2.58) = 5.49; *p* = 0.046, partial eta squared (η^2^) = 0.05. Table 3 shows the pairwise comparison for vertical jumps between the three protocols.

**Figure 2:**
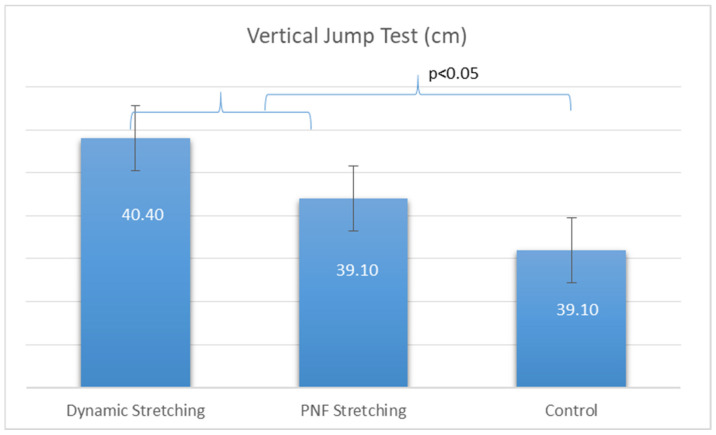
Acute effects of different stretching conditions on Vertical Jump Performance.

In the vertical jump, performance was shown to be better after dynamic stretching compared to the PNF (*p* = 0.046) and control (no stretching) group (*p* = 0.002). PNF stretching was also shown to be significantly better compared to the control (no stretching) group (*p* = 0.048) (Table 4, Figure 2).

Table 5 shows the test of the within-subject effect for the 20 m sprint. There is a significant effect in sprint performance among the three types of stretching protocol applied, F (2.58) = 5.60; *p* = 0.002, partial eta squared (η^2^) = 0.06. Table 6 shows the pairwise comparison for the 20 m sprint between the three protocols.

**Figure 3:**
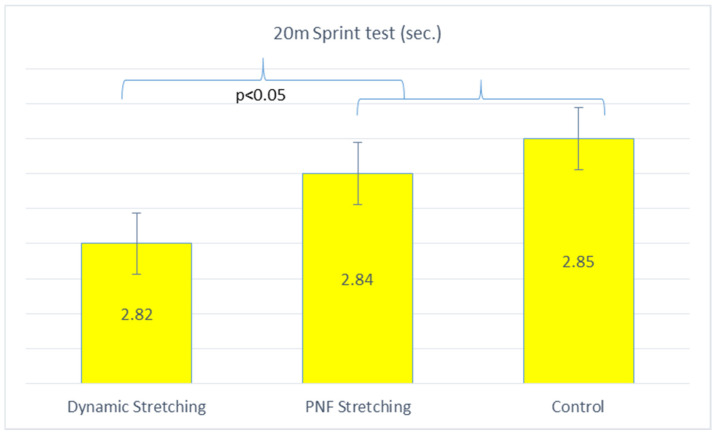
Acute effects of different stretching conditions on 20-m sprint performance.

In the 20 m sprint, performance was shown to be significantly faster after dynamic stretching compared to PNF (*p* = 0.002) and no stretching (*p* = 0.002). On the other hand, PNF stretching was shown to be better compared to no stretching (*p* = 0.049). (Table 7, Figure 3).

## 4. Discussion

The adverse effect of static stretching has been reported by several studies; thus, the implementation of dynamic and other types of stretching should be considered. The objective of this study was to examine the immediate impact of dynamic stretching, PNF stretching, and a control condition on the sprint and jump performance of recreational male individuals. Specifically, the aim was to ascertain whether there are any notable disparities in performance output and performance comparison when different types of stretching are employed prior to exercise.

The main findings of the current study were that dynamic stretching and PNF stretching lead to significant increases in sprint time and also vertical jump height compared to no stretching. This is in support of previous findings that determined these two types of stretching help in increasing the performance output of an exercise [1,2] when compared with no stretching.

When comparing the dynamic and PNF stretch protocols in this study, it is discovered that the dynamic stretching protocols showed a better performance in sprinting time (*p* = 0.002) with a difference of 0.71% faster timing than the PNF stretching protocols group. In sprinting time, 0.02 s is a big difference. Vertical jump showed a 4.40% difference (*p* = 0.046) in height, whereas the group with the dynamic warm-up protocols jumped higher than those in the PNF group. There is a difference of about 0.7 cm in height. Dynamic stretching gives an edge to the dynamic stretching group over the PNF stretching group. This result is backed by the data from numerous studies, which demonstrated an acute increase in power, sprint, or jump performance after dynamic stretches [20,24]. Dynamic stretching includes varieties of movements that are then combined with stretch movement. This type of movement, which incorporated more dynamic movement compared to PNF, can be considered as a warm-up movement by itself, thus bringing together the positive effect of a warm-up, which impacts core temperature and other temperature-related changes. Research has demonstrated that elevating muscle temperature leads to enhanced performance in activities requiring dynamic exertion over short durations [46].

PNF stretching by itself is developed to be a rehabilitation type of stretching and it is intended to be done on a person recovering from injury; thus, it requires less movement in its stretching protocol. It is somewhat similar to static stretching, but with assistance to increase flexibility. More movement protocols in dynamic stretching somewhat have a two-in-one function, promoting flexibility and elevating muscle temperature through active movement. An elevated muscle temperature usually occurs from the friction of intramuscular movement that occurs during exercise. When the muscle temperature is higher, it results in the increased transmission rate of an impulse, which is responsible for all our movement in exercise, and this will positively affect the force–velocity relationship, which is the core of performance output [46,47].

In contrast with the PNF stretching, the isometric contraction of the agonist muscle during PNF stretching serves as a key mechanism for enhancing flexibility and ROM, which may help to improve performance [48]. During proprioceptive neuromuscular facilitation (PNF) stretching, sustained isometric contraction of the stretched agonist muscle stimulates the neuromuscular spindle, leading to heightened muscle activation. This activation triggers impulses that directly influence the spinal motor neurons innervating the same muscle, intensifying the isometric contraction. Concurrently, inhibition of the antagonist muscle occurs, followed by a facilitation of the antagonist’s concentric contraction. The “reversal of antagonists” method in PNF stretching entails an extended isometric contraction of the prelengthened agonist, which is believed to release fascia tension, enhancing the muscle’s capacity to lengthen during subsequent antagonist concentric contractions. If the antagonist cannot further increase limb displacement, light pressure assists in augmenting the range of motion. Adjustment of both fascia and spindle to the new lengthened position occurs, with impulses inhibiting motor neurons to the agonist transmitted via branches. Additionally, tension elevation triggers impulses from the Golgi tendon organ (GTO), overriding neuromuscular spindle impulses, facilitating reflexive muscle relaxation (autogenic inhibition) and consequent muscle lengthening.

Even though the warm-up session is done before stretching, which resulted in a core temperature elevation, a study by Alemdaroğlu et al. [43] and Bradley et al. [29] showed that the sprint time and vertical jump height will return to normal levels after certain minutes of warming-up due to core temperature decrement. In the PNF condition, 20 m sprint performance returned to normal levels at 15 min post stretching, while 10 m performance took 20 min to recover. Bradley et al. [29] also showed a similar result, where the vertical jump performance returned to control values 15 min after stretching. Taking this into account, it can be said that the core temperature will gradually go down and this will affect performance during exercise. With this, dynamic stretching with more movement is deemed to be more useful in stretching while maintaining the core temperature at an optimum level for an exercise to produce a better performance output.

A better vertical jump height and faster sprint time when dynamic stretching is used could probably result from the effect of post-activation potentiation, which is produced from dynamic stretching [49,50,51]. The observed outcome is attributed to a temporary enhancement in muscular contractile ability subsequent to a deliberate voluntary contraction performed during the stretching process. In their study, Yamaguchi et al. [18] observed a reduction in the time required to reach peak torque and an augmentation in the rate of torque development subsequent to the implementation of dynamic stretching exercises. Based on the findings, it was determined that the occurrence of PAP is a possibility.

However, the literature also contains findings regarding compromised performance subsequent to engaging in dynamic stretching exercises [52,53,54]. However, it seems that the effect of the stretch is related and could be attributed to several factors, such as muscle group, stretching duration, stretching intensity or contraction type, and velocity. Further research on this needs to be done to add to the literature about all these determinants.

The strengths of the study were the following: comparative analysis of three groups of subjects, analysis of the impact of dynamic stretching vs. PNF on sprint and vertical jump performances; and the conception and implementation of two types of preparation protocols specific to the two types of stretching in the warm-up part. The limitations of the study were the following: the relatively small number of subjects included in the study, the non-inclusion of female subjects in the study, and the relatively short duration of the implementation of the two warm-up stretching protocols. In addition, neither core temperature nor joint range of motion was measured in this study, which is likely to be a mechanism of influencing factors on the improvement of jumping and sprinting performance.

## 5. Conclusions

Based on the aforementioned findings and the present outcomes, it can be concluded that dynamic stretches exhibit more efficacy compared to PNF stretches when employed in pre-activity warm-up routines to optimize sprint time and vertical jump height performance. There is a suggestion that including dynamic stretching into a pre-event warm-up routine may provide greater performance advantages compared to proprioceptive neuromuscular facilitation (PNF) stretching. Engaging in a brief session of dynamic stretching can lead to enhanced power output in activities such as vertical jumps and sprinting. This improvement in muscular power is notably more substantial compared to the effects of PNF stretching or the absence of stretching altogether. This knowledge may prove particularly valuable for players and coaches engaged in power-oriented sports, such as football and weightlifting.

## Figures and Tables

**Table 1:** PNF stretching protocol.

Muscle	Description
Hamstring stretch	Subject lies on their back with one leg extended and the other raised towards the ceiling. Assistant supports the raised leg and provides resistance by gently pushing the raised leg towards the subject’s face as the subject pushes against the resistance.
Quadricep stretch	Subject in prone position with one leg bent at the knee, bringing the heel towards the buttocks. Assistant supports the raised foot and provides resistance by gently pushing the foot towards the buttocks as the subject pushes against the resistance.
Groin (butterfly) stretch	Subject sits on the floor with the soles of their feet together, knees bent out to the sides. Assistant provides resistance by gently pushing the knees towards the ground as the subject pushes against the resistance.
Glute stretch	Subject in supine position. Assistant assists by gently pushing the knee of the leg being stretched towards the opposite shoulder while the other person contracts the glute by pushing the knee away from the shoulder against the resistance.

**Table 2:** Test of within-subject effect for vertical jump.

Source		Type III Sum of Square	df	Mean Square	F	Sig.	Observed Power
Test	Sphericity Assumed	25.400	2	12.700	5.49	0.046	0.253
Error (test)			58				

**Table 3:** Pairwise comparison for vertical jump (cm).

(I) Variable	(J) Variable	Mean Difference (I–J)	Std. Error	Sig.	95%CI Lower Bound	95%CI Upper Bound
Dynamic	PNF	0.700	0.990	0.046	−1.815	3.215
Dynamic	Control	1.300	1.009	0.002	−1.265	3.865
PNF	Control	0.600	0.338	0.048	−3.215	1.815

**Table 4:** Acute effects of different stretching conditions on vertical jump performance.

Group	Vertical Jump Test (cm)
Dynamic Stretching	40.40 ± 8.89 ^bc^
PNF Stretching	39.70 ± 8.82 ^c^
Control	39.10 ± 8.78 ^ab^

PNF: ^a^ significant difference from dynamic stretching; ^b^ significant difference from PNF stretching; ^c^ significant difference from control; *p* < 0.05.

**Table 5:** Test of within-subject effect for 20 m sprint (s).

Source		Type III Sum of Square	df	Mean Square	F	Sig.	Observed Power
Test	Sphericity Assumed	0.017	2	0.009	5.60	0.002	0.217
Error (test)			58				

**Table 6:** Pairwise comparison of 20 m sprint.

(I) Variable	(J) Variable	Mean Difference (I–J)	Std. Error	Sig.	95%CI Lower Bound	95%CI Upper Bound
Dynamic	PNF	−0.170	0.016	0.002	−0.057	0.023
Dynamic	Control	−0.034	0.025	0.002	−0.098	0.030
PNF	Control	−0.016	0.028	0.049	−0.088	0.056

**Table 7:** Acute effects of different stretching conditions on 20 m sprint performance.

Group	20 m Sprint Performance (s)
Dynamic Stretching	2.82 ± 0.22 ^bc^
PNF Stretching	2.84 ± 0.23 ^c^
Control	2.85 ± 0.23 ^ab^

PNF: ^a^ significant difference from dynamic stretching; ^b^ significant difference from PNF stretching; ^c^ significant difference from control; *p* < 0.05.

## Data Availability

Data are contained within the article.
